# Correction: Onvansertib in Combination with FOLFIRI and Bevacizumab in Second-Line Treatment of *KRAS*-Mutant Metastatic Colorectal Cancer: A Phase Ib Clinical Study

**DOI:** 10.1158/1078-0432.CCR-26-0622

**Published:** 2026-03-16

**Authors:** Daniel H. Ahn, Afsaneh Barzi, Maya Ridinger, Errin Samuëlsz, Ramanand A. Subramanian, Peter J.P. Croucher, Tod Smeal, Fairooz F. Kabbinavar, Heinz-Josef Lenz

In the original version of this article (1), author Chu-Chiao Wu was mistakenly omitted from the author list. This error has been corrected in the latest online HTML and PDF versions of the article and the author contributions and disclosures have been updated as necessary. The authors regret this error.
